# The Mechanism by Which Arabinoxylanases Can Recognize Highly Decorated Xylans*[Fn FN2]

**DOI:** 10.1074/jbc.M116.743948

**Published:** 2016-08-16

**Authors:** Aurore Labourel, Lucy I. Crouch, Joana L. A. Brás, Adam Jackson, Artur Rogowski, Joseph Gray, Madhav P. Yadav, Bernard Henrissat, Carlos M. G. A. Fontes, Harry J. Gilbert, Shabir Najmudin, Arnaud Baslé, Fiona Cuskin

**Affiliations:** From the ‡ Institute for Cell and Molecular Biosciences, Newcastle University, Newcastle upon Tyne NE2 4HH, United Kingdom,; § CIISA-Faculdade de Medicina Veterinária, Universidade de Lisboa, Pólo Universitário do Alto da Ajuda, Avenida da Universidade Técnica, 1300-477 Lisboa, Portugal,; ¶ NZYTech Genes & Enzymes, 1649-038 Lisboa, Portugal,; the ‖ Eastern Regional Research Center, United States Department of Agriculture-Agricultural Research Service, Wyndmoor, Pennsylvania 19038,; ** Architecture et Fonction des Macromolécules Biologiques, UMR7857 CNRS, Aix-Marseille University, F-13288 Marseille, France,; ‡‡ USC1408 Architecture et Fonction des Macromolécules Biologiques, INRA, F-13288 Marseille, France, and; the §§ Department of Biological Sciences, King Abdulaziz University, Jedda 21589, Saudi Arabia

**Keywords:** cellulosome, crystallography, enzyme kinetics, enzyme mechanism, glycoside hydrolase

## Abstract

The enzymatic degradation of plant cell walls is an important biological process of increasing environmental and industrial significance. Xylan, a major component of the plant cell wall, consists of a backbone of β-1,4-xylose (Xyl*p*) units that are often decorated with arabinofuranose (Ara*f*) side chains. A large penta-modular enzyme, *Ct*Xyl5A, was shown previously to specifically target arabinoxylans. The mechanism of substrate recognition displayed by the enzyme, however, remains unclear. Here we report the crystal structure of the arabinoxylanase and the enzyme in complex with ligands. The data showed that four of the protein modules adopt a rigid structure, which stabilizes the catalytic domain. The C-terminal non-catalytic carbohydrate binding module could not be observed in the crystal structure, suggesting positional flexibility. The structure of the enzyme in complex with Xyl*p*-β-1,4-Xyl*p*-β-1,4-Xyl*p*-[α-1,3-Ara*f*]-β-1,4-Xyl*p* showed that the Ara*f* decoration linked O_3_ to the xylose in the active site is located in the pocket (−2* subsite) that abuts onto the catalytic center. The −2* subsite can also bind to Xyl*p* and Ara*p*, explaining why the enzyme can utilize xylose and arabinose as specificity determinants. Alanine substitution of Glu^68^, Tyr^92^, or Asn^139^, which interact with arabinose and xylose side chains at the −2* subsite, abrogates catalytic activity. Distal to the active site, the xylan backbone makes limited apolar contacts with the enzyme, and the hydroxyls are solvent-exposed. This explains why *Ct*Xyl5A is capable of hydrolyzing xylans that are extensively decorated and that are recalcitrant to classic endo-xylanase attack.

## Introduction

The plant cell wall is an important biological substrate. This complex composite structure is depolymerized by microorganisms that occupy important highly competitive ecological niches, whereas the process makes an important contribution to the carbon cycle (1). Lignocellulosic degradation is also of continued interest to environmentally sensitive industries such as the biofuels and biorefinery sectors, where the use of sustainable or renewable substrates is of increasing importance. Given that the plant cell wall is the most abundant source of renewable organic carbon on the planet, this macromolecular substrate has substantial industrial potential (2).

An example of the chemical complexity of the plant cell wall is provided by xylan, which is the major hemicellulosic component. This polysaccharide comprises a backbone of β-1,4-d-xylose residues in their pyranose configuration (Xyl*p*) that are decorated at O_2_ with 4-*O*-methyl-d-glucuronic acid (GlcA) and at O_2_ and/or O_3_ with α-l-arabinofuranose (Ara*f*) residues, whereas the polysaccharide can also be extensively acetylated (3). In addition, the Ara*f* side chain decorations can also be esterified to ferulic acid that, in some species, provide a chemical link between hemicellulose and lignin (3). The precise structure of xylans varies between plant species, in particular in different tissues and during cellular differentiation (4). In specialized plant tissues, such as the outer layer of cereal grains, xylans are extremely complex, and side chains may comprise a range of other sugars including l- and d-galactose and β- and α-Xyl*p* units. Indeed, in these cereal brans, xylans have very few backbone Xyl*p* units that are undecorated, and the side chains can contain up to six sugars (5).

Reflecting the chemical and physical complexity of the plant cell wall, microorganisms that utilize these composite structures express a large number of polysaccharide-degrading enzymes, primarily glycoside hydrolases, but also polysaccharide lyases, carbohydrate esterases, and lytic polysaccharide monooxygenases. These carbohydrate active enzymes are grouped into sequence-based families in the CAZy database (6). With respect to xylan degradation, the backbone of simple xylans is hydrolyzed by endo-acting xylanases, the majority of which are located in glycoside hydrolase (GH)^5^ families GH10 and GH11, although they are also present in GH8 (1, 7). The extensive decoration of the xylan backbone generally restricts the capacity of these enzymes to attack the polysaccharide prior to removal of the side chains by a range of α-glucuronidases, α-arabinofuranosidases, and esterases (8). Two xylanases, however, utilize the side chains as essential specificity determinants and thus target decorated forms of the hemicellulose. The GH30 glucuronoxylanases require the Xyl*p* bound at the −2 to contain a GlcA side chain (9) (the scissile bond targeted by glycoside hydrolases is between subsites −1 and +1, and subsites that extend toward the non-reducing and reducing ends of the substrate are assigned increasing negative and positive numbers, respectively (10)). The GH5 arabinoxylanase (*Ct*Xyl5A) derived from *Clostridium thermocellum* displays an absolute requirement for xylans that contain Ara*f* side chains (11). In this enzyme, the key specificity determinant is the Ara*f* appended to O_3_ of the Xyl*p* bound in the active site (−1 subsite). The reaction products generated from arabinoxylans, however, suggest that Ara*f* can be accommodated at subsites distal to the active site.

**FIGURE 1.**
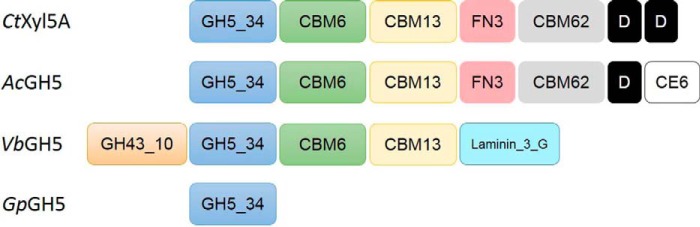
**Molecular architecture of GH5_34 enzymes.** Modules prefaced by GH, CBM, or CE are modules in the indicated glycoside hydrolase, carbohydrate binding module, or carbohydrate esterase families, respectively. Laminin_3_G domain belongs to the concanavalin A lectin superfamily, and *FN3* denotes a fibronectin type 3 domain. Segments labeled *D* are dockerin domains.

*Ct*Xyl5A is a multimodular enzyme containing, in addition to the GH5 catalytic module (*Ct*GH5); three non-catalytic carbohydrate binding modules (CBMs) belonging to families 6 (*Ct*CBM6), 13 (*Ct*CBM13), and 62 (*Ct*CBM62); fibronectin type 3 (Fn3) domain; and a C-terminal dockerin domain Fig. 1. Previous studies of Fn3 domains have indicated that they might function as ligand-binding modules, as a compact form of peptide linkers or spacers between other domains, as cellulose-disrupting modules, or as proteins that help large enzyme complexes remain soluble (12). The dockerin domain recruits the enzyme into the cellulosome, a multienzyme plant cell wall degrading complex presented on the surface of *C. thermocellum* (13, 14). *Ct*CBM6 stabilizes *Ct*GH5 (11), and *Ct*CBM62 binds to d-galactopyranose and l-arabinopyranose (15). The function of the *Ct*CBM13 and Fn3 modules remains unclear. Similarly, the mechanism of substrate recognition and its impact on specificity are key unresolved issues. This report exploits the crystal structure of mature *Ct*Xyl5A lacking its C-terminal dockerin domain (*Ct*Xyl5A_-Doc_), and the enzyme in complex with ligands, to explore the mechanism of substrate specificity. The data show that the plasticity in substrate recognition enables the enzyme to hydrolyze highly complex xylans that are not accessible to classical GH10 and GH11 endo-xylanases.

## Results

### 

#### 

##### Substrate Specificity of CtXyl5A

Previous studies showed that *Ct*Xyl5A is an arabinoxylan-specific xylanase that generates xylooligosaccharides with an arabinose linked O_3_ to the reducing end xylose (11). The enzyme is active against both wheat and rye arabinoxylans (abbreviated as WAX and RAX, respectively). It was proposed that arabinose decorations make productive interactions with a pocket (−2*) that is abutted onto the active site or −1 subsite. Arabinose side chains of the other backbone xylose units in the oligosaccharides generated by *Ct*Xyl5A were essentially random. These data suggest that O_3_, and possibly O_2_, on the xylose residues at subsites distal to the active site and −2* pocket are solvent-exposed, implying that the enzyme can access highly decorated xylans. To test this hypothesis, the activity of *Ct*Xyl5A against xylans from cereal brans was assessed. *Ct*Xyl5a was incubated with a range of xylans for 16 h at 60 °C, and the limit products were visualized by TLC. These xylans are highly decorated not only with Ara*f* and GlcA units but also with l-Gal, d-Gal, and d-Xyl (5). Indeed, very few xylose units in the backbone of bran xylans lack side chains. The data presented in Table 1 showed that *Ct*Xyl5A was active against corn bran xylan (CX). In contrast typical endo-xylanases from GH10 and GH11 were unable to attack CX, reflecting the lack of undecorated xylose units in the backbone (the active site of these enzymes can only bind to non-substituted xylose residues (8, 16)). The limit products generated by *Ct*Xyl5A from CX consisted of an extensive range of oligosaccharides. These data support the view that in subsites out with the active site the O_2_ and O_3_ groups of the bound xylose units are solvent-exposed and will thus tolerate decoration.

**TABLE 1:** **Kinetics of GH5_34 arabinoxylanases** ND, not determined; NA, no activity.

Enzyme	Variant	*k*_cat_/*K_m_*		
		WAX	RAX	CX
		*min*^−*1*^ *mg*^−*1*^ *ml*		
*Ct*Xyl5A	*Ct*GH5-CBM6-CBM13-Fn3-CBM62	800	ND	460
*Ct*Xyl5A	*Ct*GH5-CBM6-CBM13-Fn3	1,232	ND	659
*Ct*Xyl5A	*Ct*GH5-CBM6-CBM13	1,307	ND	620
*Ct*Xyl5A	*Ct*GH5-CBM6	488	ND	102
*Ct*Xyl5A	*Ct*GH5-CBM6: E68A	NA	NA	NA
*Ct*Xyl5A	*Ct*GH5-CBM6: Y92A	NA	NA	NA
*Ct*Xyl5A	*Ct*GH5-CBM6: N135A	260	ND	ND
*Ct*Xyl5A	*Ct*GH5-CBM6: N139A	NA	NA	NA
*Ac*GH5	Wild type	628	1,641	289
*Gp*GH5	Wild type	2,600	9,986	314
*Vb*GH5	Wild type	ND	ND	ND
*Vb*GH5	D45A	102	203	23

**FIGURE 2.**
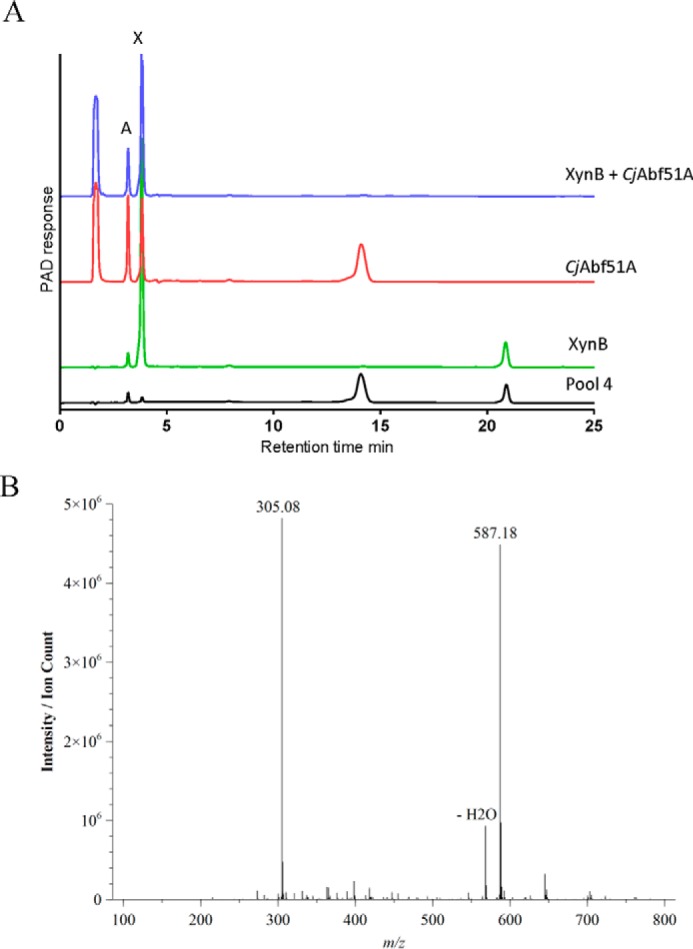
**Identification of the disaccharide reaction products generated from CX.** The smallest reaction products were purified by size exclusion chromatography and analyzed by HPAEC (*A*) and positive mode ESI-MS (*B*), respectively. The samples were treated with a nonspecific arabinofuranosidase (*Cj*Abf51A) and a GH3 xylosidase (XynB) that targeted β-1,3-xylosidic bonds. *X*, xylose; *A*, arabinose. The *m*/*z* = 305 species denotes a pentose disaccharide as a sodium adduct [M + Na]^+^, whereas the *m*/*z* = 587 signal corresponds to an ESI-MS dimer of the pentose disaccharide also as a sodium adduct [2M + Na]^+^.

To explore whether substrate bound only at −2* and −1 in the negative subsites was hydrolyzed by *Ct*Xyl5A, the limit products of CX digested by the arabinoxylanase were subjected to size exclusion chromatography using a Bio-Gel P-2, and the smallest oligosaccharides (largest elution volume) were chosen for further study. HPAEC analysis of the smallest oligosaccharide fraction (pool 4) contained two species with retention times of 14.0 min (oligosaccharide 1) and 20.8 min (oligosaccharide 2) (Fig. 2). Positive mode electrospray mass spectrometry showed that pool 4 contained exclusively molecular ions with a *m*/*z* = 305 [M + Na]^+^, which corresponds to a pentose-pentose disaccharide (molecular mass = 282 Da) as a sodium ion adduct, whereas a dimer of the disaccharide with a sodium adduct (*m*/*z* = 587 [2M+Na]^+^) was also evident. The monosaccharide composition of pool 4 determined by TFA hydrolysis contained xylose and arabinose in a 3:1 ratio. This suggests that the two oligosaccharides consist of two disaccharides: one consisting of two xylose residues and the other consisting of an arabinose linked to a xylose. Treatment of pool 4 with the nonspecific arabinofuranosidase, *Cj*Abf51A (17), resulted in the loss of oligosaccharide 2 and the production of both xylose and arabinose, indicative of a disaccharide of xylose and arabinose. Incubation of pool 4 with a β-1,3-xylosidase (XynB) converted oligosaccharide 1 into xylose, demonstrating that this molecule is the disaccharide β-1,3-xylobiose. This view is supported by the inability of a β-1,4-specific xylosidase to hydrolyze oligosaccharide 1 or oligosaccharide 2 (data not shown). The crucial importance of occupancy of the −2* pocket for catalytic competence is illustrated by the inability of the enzyme to hydrolyze linear β-1,4-xylooligosaccharides. The generation of Ara*f*-Xyl*p* and Xyl-β-1,3-Xyl as reaction products demonstrates that occupancy of the −2 subsite is not essential for catalytic activity, which is in contrast to all endo-acting xylanases where this subsite plays a critical role in enzyme activity (18, 19). Indeed, the data demonstrate that −2* plays a more important role in productive substrate binding than the −2 subsite. Unfortunately, the inability to generate highly purified (Xyl-β-1,4)*_n_*-[β-1,3-Xyl/Ara]-Xyl oligosaccharides from arabinoxylans prevented the precise binding energies at the negative subsites to be determined.

##### Crystal Structure of the Catalytic Module of CtXyl5A in Complex with Ligands

**FIGURE 3.**
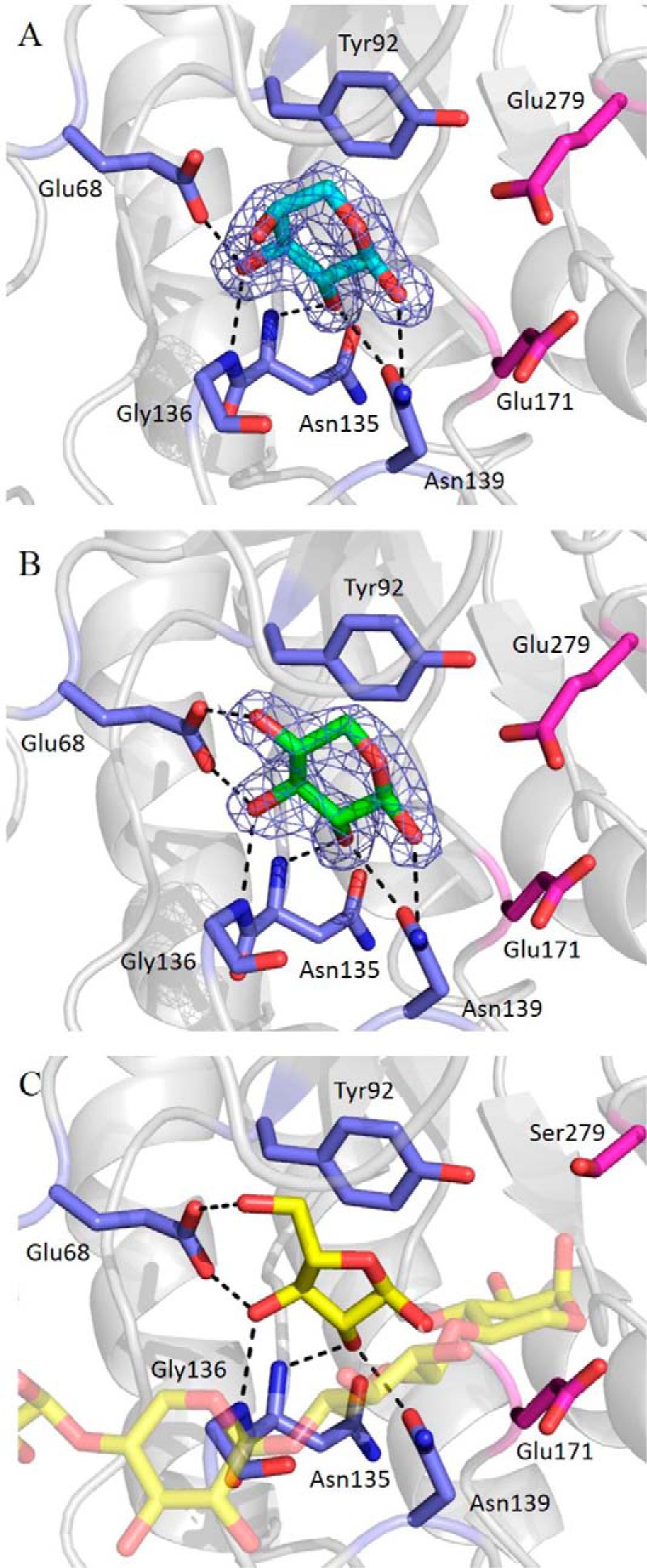
Representation of the residues involved in the ligands recognition at the −2* subsite. The protein backbone is represented as a cartoon in *gray*. Interacting residues are represented as stick in *blue*, and the catalytic residues and the mutated glutamate (into a serine) are in *magenta. A*, *Ct*GH5-CBM6 in complex with an arabinopyranose. *B*, *Ct*GH5-CBM6 in complex with a xylopyranose. *C*, *Ct*GH5_E279S_-CBM6 in complex with a pentasaccharide (β1,4-xylotetraose with an l-Ara*f* linked α1,3 to the reducing end xylose). The xylan backbone is shown transparently for more clarity. Densities shown in *blue* are RefMac maximum-likelihood σ_A_-weighted 2*F*_o_ − *F*_c_ at 1.5 σ. The figure and all other structural figures were made with PyMOL unless otherwise stated.

To understand the structural basis for the biochemical properties of *Ct*Xyl5A, the crystal structure of the enzyme with ligands that occupy the substrate binding cleft and the critical −2* subsite were sought. The data presented in Fig. 3*A* show the structure of the *Ct*Xyl5A derivative *Ct*GH5-*Ct*CBM6 in complex with arabinose bound in the −2* pocket. Interestingly, the bound arabinose was in the pyranose conformation rather than in its furanose form found in arabinoxylans. O_1_ was facing toward the active site −1 subsite, indicative of the bound arabinose being in the right orientation to be linked to the xylan backbone via an α-1,3 linkage. As discussed on below, the axial O_4_ of the Ara*p* did not interact with the −2* subsite, suggesting that the pocket might be capable of binding a xylose molecule. Indeed, soaking apo crystals with xylose showed that the pentose sugar also bound in the −2* subsite in its pyranose conformation (Fig. 3*B*). These crystal structures support the biochemical data presented above showing that the enzyme generated β-1,3-xylobiose from CX, which would require the disaccharide to bind at the −1 and −2* subsites. A third product complex was generated by co-crystallizing the nucleophile inactive mutant *Ct*GH5_E279S_-*Ct*CBM6 with a WAX-derived oligosaccharide (Fig. 3*C*). The data revealed a pentasaccharide bound to the enzyme, comprising β-1,4-xylotetraose with an Ara*f* linked α-1,3 to the reducing end xylose. The xylotetraose was positioned in subsites −1 to −4 and the Ara*f* in the −2* pocket. Analysis of the three structures showed that O_1_, O_2_, O_3_, and the endocyclic oxygen occupied identical positions in the Ara*p*, Ara*f*, and Xyl*p* ligands bound in the −2* subsite and thus made identical interactions with the pocket. O_1_ makes a polar contact with Nδ2 of Asn^139^, O_2_ is within hydrogen bonding distance with Oδ1 of Asn^139^ and the backbone N of Asn^135^, and O_3_ interacts with the N of Gly^136^ and Oϵ2 of Glu^68^. Although O_4_ of Ara*p* does not make a direct interaction with the enzyme, O_4_ and O_5_ of Xyl*p* and Ara*f*, respectively, form hydrogen bonds with Oϵ1 of Glu^68^. Finally Tyr^92^ makes apolar parallel interactions with the pyranose or furanose rings of the three sugars.

The importance of the interactions between the ligands and the side chains of the residues in the −2* pocket were evaluated by alanine substitution of these amino acids. The mutants E68A, Y92A, and N139A were all inactive (Table 1), demonstrating the importance of the interactions of these residues with the substrate and reinforcing the critical role the −2* subsite plays in the activity of the enzyme. N135A retained wild type activity because the O_2_ of the sugars interacts with the backbone N of Asn^135^ and not with the side chain. Because the hydroxyls of Xyl*p* or Ara*f* in the −2* pocket are not solvent-exposed, the active site of the arabinoxylanase can only bind to xylose residues that contain a single xylose or arabinose O_3_ decoration. This may explain why the *k*_cat_/*K_m_* for *Ct*Xyl5A against WAX was 2-fold higher than against CX (Table 1). WAX is likely to have a higher concentration of single Ara*f* decorations compared with CX and thus contain more substrate available to the arabinoxylanase.

**FIGURE 4.**
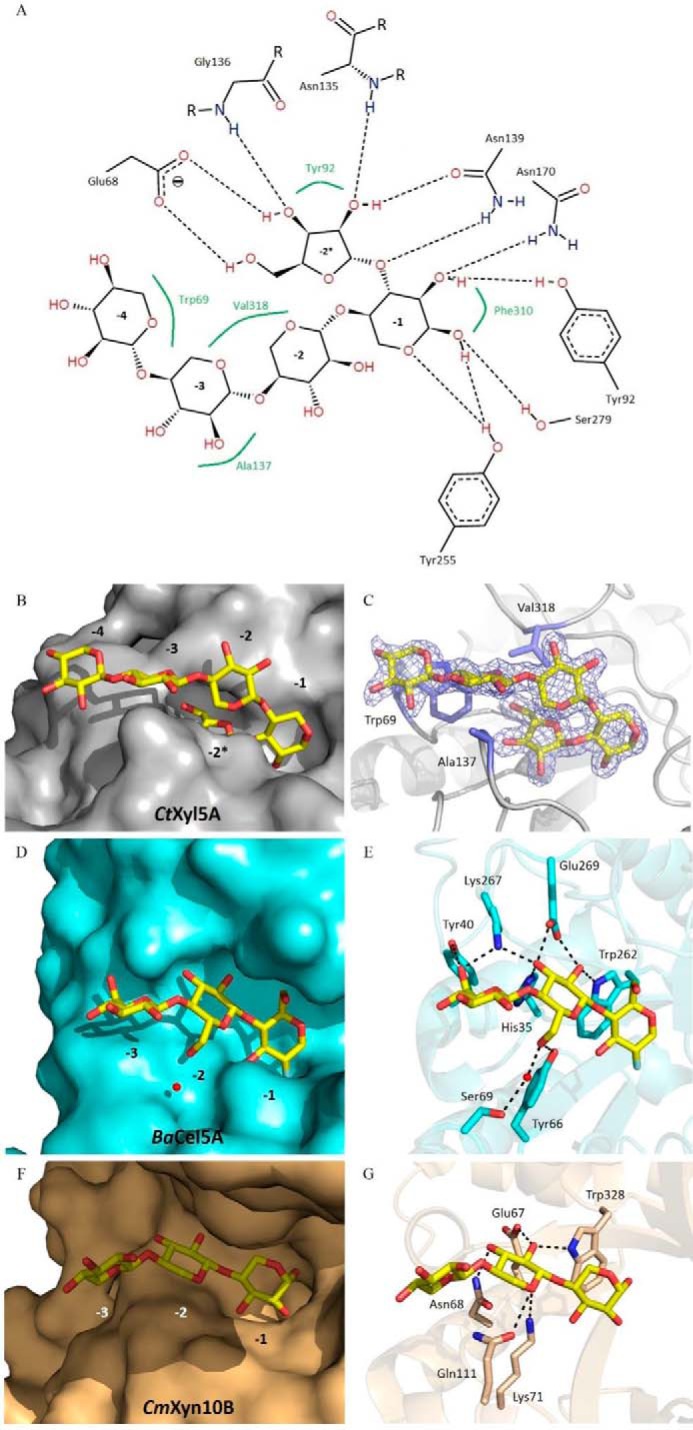
**Comparison of the ligand recognition at the distal negative subsites between *Ct*GH5_E279S_-CBM6, the cellulase *Ba*Cel5A, and the xylanase GH10.** *A–C* show *Ct*GH5_E279S_-CBM6 is in complex with a pentasaccharide (β1,4-xylotetraose with an l-Ara*f* linked α1,3 to the reducing end xylose). *A*, Poseview (40) representation highlighting the hydrogen bonding and the hydrophobic interactions that occur in the negative subsites. *C*, density of the ligand shown in *blue* is RefMac maximum-likelihood σ_A_-weighted 2*F*_o_ − *F*_c_ at 1.5 σ. *D* and *E* display *Ba*Cel5A in complex with deoxy-2-fluoro-β-d-cellotrioside (PDB code 1qi2), and *F* and *G* show *Cm*Xyn10B in complex with a xylotriose (PDB code 1uqy). The ligand are represented as *sticks. B*, *D*, and *F* are surface representations (*Ct*GH5_E279S_-CBM6 in *gray*, *Ba*Cel5A in *cyan*, and the xylanase GH10 in *light brown*). *C*, *E*, and *G* show the protein backbone as a cartoon representation with the interacting residues represented as *sticks*. The *black dashes* represent the hydrogen bonds.

In the active site of *Ct*Xyl5A the α-d-Xyl*p*, which is in its relaxed ^4^C_1_ conformation, makes the following interactions with the enzyme (Fig. 4, *A–C*): O_1_ hydrogen bonds with the Nδ1 of His^253^ and Oϵ2 of Glu^171^ (catalytic acid-base) and makes a possible weak polar contact with the OH of Tyr^255^ and Oγ of Ser^279^ (mutation of the catalytic nucleophile); O_2_ hydrogen bonds with Nδ2 of Asn^170^ and OH of Tyr^92^. O_3_ (O_1_ of the Ara*f* at the −2* subsite) makes a polar contact with Nδ2 of Asn^139^; the endocyclic oxygen hydrogens bonds with the OH of Tyr^255^. The Xyl*p* in the active site makes strong parallel apolar interactions with Phe^310^. Substrate recognition in the active site is conserved between *Ct*Xyl5A and the closest GH5 structural homolog, the endoglucanase *Ba*Cel5A (PDB code 1qi2) as noted previously (11).

The capacity of *Ct*Xyl5A to act on the highly decorated xylan CX indicates that O_3_ and possibly O_2_ of the backbone Xyl*p* units are solvent-exposed. This is consistent with the interaction of the xylotetraose backbone with the enzyme distal to the active site. A surface representation of the enzyme (Fig. 4*B*) shows that O_3_ and O_2_ of xylose units at subsites −2 to −4 are solvent-exposed and are thus available for decoration. Indeed, these pyranose sugars make very weak apolar interactions with the arabinoxylanase. At −2, Xyl*p* makes planar apolar interactions with the Ara*f* bound to the −2* subsite (Fig. 4*C*). Xyl*p* at subsites −2 and −3, respectively, make weak hydrophobic contact with Val^318^, the −3 Xyl*p* makes planar apolar interactions with Ala^137^, whereas the xylose at −4 forms parallel apolar contacts with Trp^69^. Comparison of the distal negative subsites of *Ct*Xyl5A with *Ba*Cel5A and a typical GH10 xylanase (*Cm*Xyn10B, PDB code 1uqy) highlights the paucity of interactions between the arabinoxylanase and its substrate out with the active site (Fig. 4). Thus, the cellulase contains three negative subsites and the sugars bound in the −2 and −3 subsites make a total of 9 polar interactions with the enzyme (Fig. 4, *D* and *E*). The GH10 xylanase also contains a −2 subsite that, similar to the cellulase, makes numerous interactions with the substrate (Fig. 4, *F* and *G*).

##### The Influence of the Modular Architecture of CtXyl5A on Catalytic Activity

*Ct*Xyl5A, in addition to its catalytic module, contains three CBMs (*Ct*CBM6, *Ct*CBM13, and *Ct*CBM62) and a fibronectin domain (*Ct*Fn3). A previous study showed that although the CBM6 bound in an exo-mode to xylo- and cellulooligosaccharides, the primary role of this module was to stabilize the structure of the GH5 catalytic module (11). To explore the contribution of the other non-catalytic modules to *Ct*Xyl5A function, the activity of a series of truncated derivatives of the arabinoxylanase were assessed. The data in Table 1 show that removal of *Ct*CBM62 caused a modest increase in activity against both WAX and CX, whereas deletion of the Fn3 domain had no further impact on catalytic performance. Truncation of *Ct*CBM13, however, caused a 4–5-fold reduction in activity against both substrates. Members of CBM13 have been shown to bind to xylans, mannose, and galactose residues in complex glycans (20–23), hinting that the function of *Ct*CBM13 is to increase the proximity of substrate to the catalytic module of *Ct*Xyl5A. Binding studies, however, showed that *Ct*CBM13 displayed no affinity for a range of relevant glycans including WAX, CX, xylose, mannose, galactose, and birchwood xylan (BX) (data not shown). It would appear, therefore, that *Ct*CBM13 makes a structural contribution to the function of *Ct*Xyl5A.

##### Crystal Structure of CtXyl5A_-D_

**FIGURE 5.**
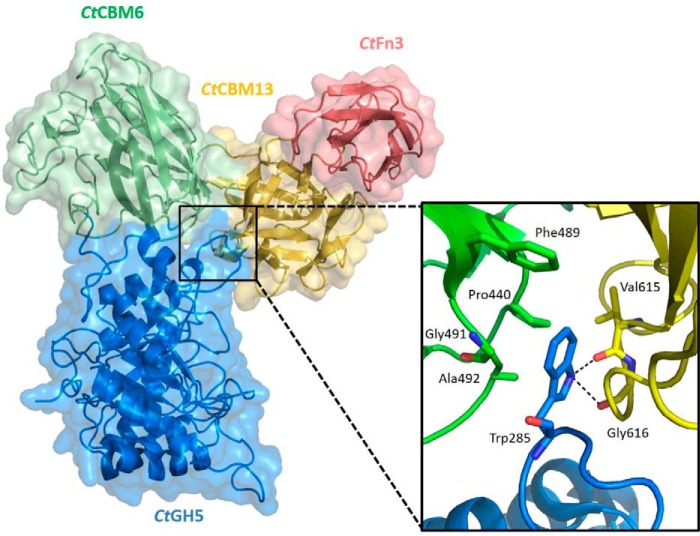
**Surface representation of the tetra-modular arabinoxylanase and zoom view on the *Ct*GH5 loop.** The *blue* module is the *Ct*GH5 catalytic domain, the *green* module corresponds to the *Ct*CBM6, the *yellow* module is the *Ct*CBM13, and the *salmon* module is the fibronectin domain. Surfaces are semitransparent with the protein backbone represented as a cartoon. The *Ct*GH5 loop is stabilized between the *Ct*CBM6 and the *Ct*CBM13 modules. The *black dashes* represent the hydrogen bonds. The protein backbone is represented as cartoon, and interacting residues are shown as *sticks*.

To explore further the role of the non-catalytic modules in *Ct*Xyl5A the crystal structure of *Ct*Xyl5A extending from *Ct*GH5 to *Ct*CBM62 was sought. To obtain a construct that could potentially be crystallized, the protein was generated without the C-terminal dockerin domain because it is known to be unstable and prone to cleavage. Using this construct (*Ct*Xyl5A_-D_) the crystal structure of the arabinoxylanase was determined by molecular replacement to a resolution of 2.64 Å with *R*_work_ and *R_free_* at 23.7% and 27.8%, respectively. The structure comprises a continuous polypeptide extending from Ala^36^ to Trp^742^ displaying four modules GH5-CBM6-CBM13-Fn3. Although there was some electron density for *Ct*CBM62, it was not sufficient to confidently build the module (Fig. 5). Further investigation of the crystal packing revealed a large solvent channel adjacent to the area the CBM62 occupies. We postulate that the reason for the poor electron density is due to the *Ct*CBM62 being mobile compared with the rest of the protein. The structures of *Ct*GH5 and *Ct*CBM6 have been described previously (11, 15).

*Ct*CBM13 extends from Gly^567^ to Pro^648^. Typical of CBM13 proteins *Ct*CBM13 displays a β-trefoil fold comprising the canonical pseudo 3-fold symmetry with a 3-fold repeating unit of 40–50 amino acid residues characteristic of the Ricin superfamily. Each repeat contains two pairs of antiparallel β-strands. A Dali search revealed structural homologs from the CBM13 family with an root mean square deviation less than 2.0 Å and sequence identities of less than 20% that include the functionally relevant homologs *C. thermocellum* exo-β-1,3-galactanase (22) (PDB code 3vsz), *Streptomyces avermitilis* β-l-arabinopyranosidase (21) (PDB code 3a21), *Streptomyces lividans* xylanase 10A (23) (PDB code, 1mc9), and *Streptomyces olivaceoviridis* E-86 xylanase 10A (20) (PDB code 1v6v).

The Fn3 module displays a typical β-sandwich fold with the two sheets comprising, primarily, three antiparallel strands in the order β1-β2-β5 in β-sheet 1 and β4-β3-β6 in β-sheet 2. Although β-sheet 2 presents a cleft-like topology, typical of endo-binding CBMs, the surface lacks aromatic residues that play a key role in ligand recognition, and in the context of the full-length enzyme, the cleft abuts into *Ct*CBM13 and thus would not be able to accommodate an extended polysaccharide chain (see below).

In the structure of *Ct*Xyl5A_-D_, the four modules form a three-leaf clover-like structure (Fig. 5). Between the interfaces of *Ct*GH5-CBM6-CBM13 there are a number of interactions that maintain the modules in a fixed position relative to each other. The interaction of *Ct*GH5 and *Ct*CBM6, which buries a substantial apolar solvent-exposed surface of the two modules, has been described previously (11). The polar interactions between these two modules comprise 14 hydrogen bonds and 5 salt bridges. The apolar and polar interactions between these two modules likely explaining why they do not fold independently compared with other glycoside hydrolases that contain CBMs (24, 25). *Ct*CBM13 acts as the central domain, which interacts with *Ct*GH5, *Ct*CBM6, and *Ct*Fn3 via 2, 5, and 4 hydrogen bonds, respectively, burying a surface area of ∼450, 350, and 500 Å^2^, respectively, to form a compact heterotetramer. With respect to the *Ct*CBM6-CBM13 interface, the linker (SPISTGTIP) between the two modules, extending from Ser^514^ to Pro^522^, adopts a fixed conformation. Such sequences are normally extremely flexible (26); however, the two Ile residues make extensive apolar contacts within the linker and with the two CBMs, leading to conformational stabilization. The interactions between *Ct*GH5 and the two CBMs, which are mediated by the tip of the loop between β-7 and α-7 (loop 7) of *Ct*GH5, not only stabilize the trimodular clover-like structure but also make a contribution to catalytic function. Central to the interactions between the three modules is Trp^285^, which is intercalated between the two CBMs. The Nϵ of this aromatic residue makes hydrogen bonds with the backbone carbonyl of Val^615^ and Gly^616^ in *Ct*CBM13, and the indole ring makes several apolar contacts with *Ct*CBM6 (Pro^440^, Phe^489^, Gly^491^, and Ala^492^) (Fig. 5). Indeed, loop 7 is completely disordered in the truncated derivative of *Ct*Xyl5A comprising *Ct*GH5 and *Ct*CBM6, demonstrating that the interactions with *Ct*CBM13 stabilize the conformation of this loop. Although the tip of loop 7 does not directly contribute to the topology of the active site, it is only ∼12 Å from the catalytic nucleophile Glu^279^. Thus, any perturbation of the loop (through the removal of *Ct*CBM13) is likely to influence the electrostatic and apolar environment of the catalytic apparatus, which could explain the reduction in activity associated with the deletion of *Ct*CBM13.

Similar to the interactions between *Ct*CBM6 and *Ct*CBM13, there are extensive hydrophobic interactions between *Ct*CBM13 and *Ct*Fn3, resulting in very little flexibility between these modules. As stated above, the absence of *Ct*CBM62 in the structure suggests that the module can adopt multiple positions with respect to the rest of the protein. The *Ct*CBM62, by binding to its ligands (d-Gal*p* and l-Ara*p*) in plant cell walls (15), may be able to recruit the enzyme onto its target substrate. Xylans are not generally thought to contain such sugars. d-Gal*p*, however, has been detected in xylans in the outer layer of cereal grains and in eucalyptus trees (5), which are substrates used by *Ct*Xyl5A. Thus, *Ct*CBM62 may direct the enzyme to particularly complex xylans containing d-Gal*p* at the non-reducing termini of the side chains, consistent with the open substrate binding cleft of the arabinoxylanase that is optimized to bind highly decorated forms of the hemicellulose. In general CBMs have little influence on enzyme activity against soluble substrates but have a significant impact on glycans within plant cell walls (27, 28). Thus, the role of CBM62 will likely only be evident against insoluble composite substrates.

##### Exploring GH5 Subfamily 34

**FIGURE 6.**
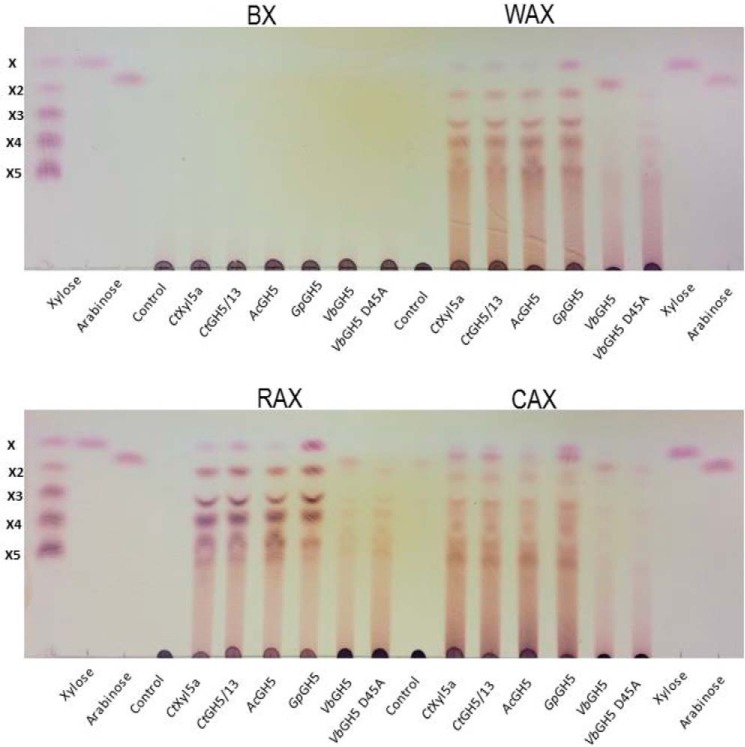
**Products profile generated of GH5_34 enzymes.** The enzymes at 1 μm were incubated with the four different xylans at 1% in 50 mm sodium phosphate buffer for 16 h at 37 °C (*Gp*GH5, *Vb*GH5, and *Ac*GH5) or 60 °C. The limit products were separated by TLC. The xylooligosaccharide standards (*X*) are indicated by their degrees of polymerization.

*Ct*Xyl5A is a member of a seven-protein subfamily of GH5, GH5_34 (29). Four of these proteins are distinct, whereas the other three members are essentially identical (derived from different strains of *C. thermocellum*). To investigate further the substrate specificity within this subfamily, recombinant forms of three members of GH5_34 that were distinct from *Ct*Xyl5A were generated. *Ac*GH5 has a similar molecular architecture to *Ct*Xyl5A with the exception of an additional carbohydrate esterase family 6 module at the C terminus (Fig. 1). The GH5_34 from *Verrucomicrobiae bacterium*, *Vb*GH5, contains the GH5-CBM6-CBM13 core structure, but the C-terminal Fn3-CBM62-dockerin modules, present in *Ct*Xyl5A, are replaced with a Laminin_3_G domain, which, by analogy to homologous domains in other proteins that have affinity for carbohydrates (30), may display a glycan binding function. The *Verrucomicobiae* enzyme also has an N-terminal GH43 subfamily 10 (GH43_10) catalytic module. The fungal GH5_34, *Gp*GH5, unlike the two bacterial homologs, comprises a single GH5 catalytic module lacking all of the other accessory modules (Fig. 1). *Gp*Gh5 is particularly interesting as *Gonapodya prolifera* is the only fungus of the several hundred fungal genomes that encodes a GH5_34 enzyme. In fact there are four potential GH5_34 sequences in the *G. prolifera* genome, all of which show high sequence homology to *Clostridium* GH5_34 sequences. *G. prolifera* and *Clostridium* occupy similar environments, suggesting that the *Gp*GH5_34 gene was acquired from a *Clostridium* species, which was followed by duplication of the gene in the fungal genome. The sequence identity of the GH5_34 catalytic modules with *Ct*Xyl5A ranged from 55 to 80% (supplemental Fig. S1). All the GH5_34 enzymes were active on the arabinoxylans RAX, WAX, and CX but displayed no activity on BX (Table 1 and Fig. 6) and are thus defined as arabinoxylanases. The limit products generated by *Ct*Xyl5A, *Ac*GH5, and *Gp*GH5 comprised a range of oligosaccharides with some high molecular weight material. The oligosaccharides with low degrees of polymerization were absent in the *Vb*GH5 reaction products. However, the enzyme generated a large amount of arabinose, which was not produced by the other arabinoxylanases. Given that GH43_10 is predominantly an arabinofuranosidase subfamily of GH43 (31), the arabinose generated by *Vb*GH5 is likely mediated by the N-terminal catalytic module (see below). Kinetic analysis showed that *Ac*GH5 displayed similar activity to *Ct*Xyl5A against both WAX and RAX and was 2-fold less active against CX. When initially measuring the activity of wild type *Vb*GH5 against the different substrates, no clear data could be obtained, regardless of the concentration of enzyme used the reaction appeared to cease after a few minutes. We hypothesized that the N-terminal GH43_10 rapidly removed single arabinose decorations from the arabinoxylans depleting the substrate available to the arabinoxylanase, explaining why this activity was short lived. To test this hypothesis, the conserved catalytic base (Asp^45^) of the GH43_10 module of *Vb*GH5 was substituted with alanine, which is predicted to inactivate this catalytic module. The D45A mutant did not produce arabinose consistent with the arabinofuranosidase activity displayed by the GH43_10 module in the wild type enzyme (Fig. 6). The kinetics of the GH5_34 arabinoxylanase catalytic module was now measurable, and activities were determined to be between ∼6- and 10-fold lower than that of *Ct*Xyl5A. Interestingly, the fungal arabinoxylanase displays the highest activities against WAX and RAX, ∼4- and 6-fold higher, respectively, than *Ct*Xyl5A; however, there is very little difference in the activity between the eukaryotic and prokaryotic enzymes against CX. Attempts to express individual modules of a variety of truncations of *Ac*GH5 and *Vb*GH5 were unsuccessful. This may indicate that the individual modules can only fold correctly when incorporated into the full-length enzyme, demonstrating the importance of intermodule interactions to maintain the structural integrity of these enzymes.

## Discussion

A characteristic feature of enzymes that attack the plant cell wall is their complex molecular architecture (1). The CBMs in these enzymes generally play a role in substrate targeting (25, 28) and are appended to the catalytic modules through flexible linker sequences (26). *Ct*Xyl5A provides a rare visualization of the structure of multiple modules within a single enzyme. The central feature of these data is the structural role played by two of the CBMs, *Ct*CBM6 and *Ct*CBM13, in maintaining the active conformation of the catalytic module, *Ct*GH5. The crystallographic data described here are supported by biochemical data showing either that these two modules do not bind to glycans (*Ct*CBM13) or that the recognition of the non-reducing end of xylan or cellulose chains (*Ct*CBM6) is unlikely to be biologically significant. It should be emphasized, however, that glycan binding and substrate targeting may only be evident in the full-length enzyme acting on highly complex structures such as the plant cell wall, as observed recently by a CBM46 module in the *Bacillus* xyloglucanase/mixed linked glucanase *Bh*Cel5B (27).

*Ct*Xyl5A is a member of GH5 that contains 6644 members. These proteins have been subdivided into 51 subfamilies based on sequence similarity (29). *Ct*Xyl5A is a member of subfamily GH5_34. Here we have explored the substrate specificity of the other members of this subfamily. Despite differences in sequence identity all of the homologs were shown to be arabinoxylanases. Consistent with the conserved substrate specificity, all members of GH5_34 contained the specificity determinants Glu^68^, Tyr^92^, and Asn^139^, which make critical interactions with the xylose or arabinose in the −2* subsite, which are 1,3-linked to the xylose positioned in the active site. The presence of a CBM62 in *Ct*Xyl5A and *Ac*GH5 suggests that these enzymes target highly complex xylans that contain d-galactose in their side chains. The absence of a “non-structural” CBM in *Gp*GH5 may indicate that this arabinoxylanase is designed to target simpler arabinoxylans present in the endosperm of cereals. Although the characterization of all members of GH5_34 suggests that this subfamily is monospecific, differences in specificity are observed in other subfamilies of GHs including GH43 (31) and GH5 (29). Thus, as new members of GH5_34 are identified from genomic sequence data and subsequently characterized, the specificity of this family may require reinterpretation.

An intriguing feature of *Vb*GH5 is that the limited products generated by this enzymes are much larger than those produced by the other arabinoxylanases. This suggests that although arabinose decorations contribute to enzyme specificity (*Vb*GH5 is not active on xylans lacking arabinose side chains), the enzyme requires other specificity determinants that occur less frequently in arabinoxylans. This has some resonance with a recently described GH98 xylanase that also exploits specificity determinants that occur infrequently and are only evident in highly complex xylans (*e.g.* CX) (5).

To conclude, this study provides the molecular basis for the specificity displayed by arabinoxylanases. Substrate specificity is dominated by the pocket that binds single arabinose or xylose side chains. The open xylan binding cleft explains why the enzyme is able to attack highly decorated forms of the hemicellulose. It is also evident that appending additional catalytic modules and CBMs onto the core components of these enzymes generates bespoke arabinoxylanases with activities optimized for specific functions. The specificities of the arabinoxylanases described here are distinct from the classical endo-xylanases and thus have the potential to contribute to the toolbox of biocatalysts required by industries that exploit the plant cell wall as a sustainable substrate.

## Experimental Procedures

### 

#### 

##### Cloning, Expression, and Purification of Components of CtXyl5A

All recombinant forms of *Ct*Xyl5A used in this study were expressed in the cytoplasm of *Escherichia coli* because they lacked a signal peptide. DNA encoding *Ct*GH5-*Ct*CBM6 and *Ct*Xyl5A_-D_ (*Ct*Xyl5A lacking the C-terminal dockerin domain (*Ct*GH5-*Ct*CBM6*-Ct*CBM13-Fn3*-Ct*CBM62)) were described previously (11). DNA encoding *Ct*GH5-*Ct*CBM6*-Ct*CBM13-Fn3 and *Ct*GH5-*Ct*CBM6*-Ct*CBM13 and mature *Acetivibrio cellulolyticus* GH5 (*Ac*GH5) were amplified by PCR using plasmid encoding the full-length *C. thermocellum* arabinoxylanase or *A. cellulolyticus* genomic DNA as the respective templates. DNA encoding the *G. prolifera* GH5 (*Gp*GH5) and *V. bacterium* GH5 (*Vb*GH5) were initially generated by GeneArt® gene synthesis (Thermo Fisher Scientific). DNA encoding *Vb*GH5 lacking the C-terminal cell surface anchoring residues was also amplified by PCR using the synthesized nucleic acid as the template. All the primers used in the PCRs required restriction sites and plasmids used are listed inj supplemental Table S1. All constructs were cloned such that the encoded proteins contain a C-terminal His_6_ tag. Site-directed mutagenesis was carried out using the PCR-based QuikChange method (Stratagene) deploying the primers listed in supplemental Table S1.

To express the recombinant proteins, *E. coli* strain BL21(DE3), harboring appropriate recombinant plasmids, was cultured to mid-exponential phase in Luria broth at 37 °C. Isopropyl β-d-galactopyranoside at 1 mm was then added to induce recombinant gene expression, and the culture incubated for a further 18 h at 16 °C. The recombinant proteins were purified to >90% electrophoretic purity by immobilized metal ion affinity chromatography using Talon^TM^ (Clontech), cobalt-based matrix, and elution with 100 mm imidazole, as described previously (33). When preparing the selenomethionine derivative of *Ct*Xyl5A_-D_ for crystallography, the proteins were expressed in *E. coli* B834 (DE3), a methionine auxotroph, cultured in medium comprising 1 liter of SelenoMet Medium Base^TM^, 50 ml of SelenoMet^TM^ nutrient mix (Molecular Dimensions), and 4 ml of a 10 mg/ml solution of l-selenomethionine. Recombinant gene expression and protein purification were as described above except that all purification buffers were supplemented with 10 mm β-mercaptoethanol.

##### Enzyme Assays

*Ct*Xyl5A_-D_ and its derivatives were assayed for enzyme activity using the method of Miller (34) to detect the release of reducing sugar. The standard assay was carried out in 50 mm sodium phosphate buffer, pH 7.0, containing 0.1 mg/ml BSA and at substrate concentrations ranging from 1 to 6 mg/ml. The pH and temperature optima were previously determined to be 7 and 60 °C, respectively, for the *Ct*Xyl5A_-D_ and its derivatives. The optimum temperature for the other enzymes was found to be 37 °C, and pH optima of 5, 7, and 4 were determined for *Ac*GH5, *Gp*GH5 and *Vb*GH5, respectively. All enzymes were assayed for activity at their individual temperature and pH optimum. A FLUOstar Omega microplate reader (BMG Labtech) was used to measure activity in 96-well plates. Overnight assays to assess end point products were carried out with 6 mg/ml substrate and 1 μm enzyme concentrations. The identification of potential reaction products was also assessed by HPAEC or TLC using methodology described previously (34).

##### Oligosaccharide Analysis

Approximately 5 g of CX or WAX were digested to completion (no further increase in reducing sugar and change in the HPAEC product profile) with 3 μm of *Ct*Xyl5A_-D_ at 60 °C for 48 h. The oligosaccharide products were purified by size exclusion chromatography using a Bio-Gel P2 column as described previously (35). The structures of the oligosaccharides were analyzed by positive ion-mode infusion/offline electrospray ionization (ESI)-MS following either dilution with 30% acetonitrile or via desalting as described previously (36)

##### Crystallography

Purified SeMet *Ct*Xyl5A_-D_ was concentrated and stored in 5 mm DTT, 2 mm CaCl_2_. Crystals of seleno-l-methionine-containing protein were obtained by hanging drop vapor diffusion in 40% (v/v) 2-methyl-2,4-pentandiol. The data were collected on Beamlines ID14-1 and ID14-4 at the European Synchrotron Radiation Facility (Grenoble, France) to a resolution of 2.64 Å. The data were processed using the programs iMOSFLM (37) and SCALA (38) from the CCP4 suite (Collaborative Computational Project, Number 4, 1994). The crystal belongs to the orthorhombic space group (P2_1_2_1_2) (39). The structure was solved by molecular replacement using independently solved structures of some of the modules of the *Ct*Xyl5A: *Ct*GH5-CBM6 (PDB code 2y8k) (11), Fn3 (PDB code 3mpc) (12), and *Ct*CBM62 (PDB codes 2y8m, 2yfz, and 2y9s) (15) using PHASER (41). The *Ct*CBM13 domain was built *de novo*. BUCCANEER (42) and PHENIX (43) were initially used for auto building. The structure was completed by iterative cycles of manual rebuilding in COOT (44) in tandem with refinement with RefMac5 (45). The final values for *R*_work_ and *R*_free_) were 23.73 and 27.80%) using TLS and restraining refinement to amino acid residues 36–373 representing the *Ct*GH5 module, 374–516 for the *Ct*CBM6, 517–652 for *Ct*CBM13, and 653–742 for *Ct*Fn3. Stereochemistry was assessed with COOT (44) and PDBSUM (46) (with 677 residues (96%) in preferred, 22 in allowed regions (3%), and 6 outliers (1%) in the Ramachandran plot).

To obtain structures of *Ct*GH5-CBM6 in complex with ligand the protein was crystallized using the sitting drop vapor phase diffusion method with an equal volume (100 nl) of protein and reservoir solution (unless otherwise stated), using the robotic nanodrop dispensing systems (mosquito^R^ LCP; TTPLabTech). Crystals of the protein (10 mg/ml) co-crystallized with arabinose (300 mm) were obtained in 1 m ammonium sulfate, 0.1 m Bis-Tris, pH 5.5, and 1% PEG 3350. Crystals with xylose (300 mm) grew in 100 mm sodium/potassium phosphate, 100 mm MES, pH 6.5, and 2 m sodium chloride. To obtain crystals of the arabinoxylanase in complex with an oligosaccharide, the nucleophile mutant E279S was used and mixed with a range of arabinoxylooligosaccharides that was generated by digestion of WAX with *Ct*GH5-CBM6 (see above) and thereafter by 100 nm of the *Cellvibrio japonicus* GH43 exo-1,4-β-xylosidase (47). Only the inclusion of the largest purified oligosaccharide generated crystals of the arabinoxylanase. Crystals of *Ct*GH5_E279S_-CBM6 were obtained by mixing an equal volume (100 nl) of the protein (11 mg/ml)/oligosaccharide (10 mm) solution and mother liquor solution consisting of 100 mm Tris-Bicine, pH 8.5, 12.5% (w/v) polyethylene glycol with an average molecular mass of 1,000 Da, 12.5% (w/v) polyethylene glycol with an average molecular mass of 3,350 Da and 12.5% (*R*,*S*)-2-methyl-2,4-pentanediol (racemic). Crystallographic data were collected on Beamlines IO2, IO4-1, and I24 at the DIAMOND Light Source (Harwell, UK). The data were processed using XDS (48) The crystal structures were solved by molecular replacement using MolRep (49) with *Ct*GH5-*Ct*CBM6 (PDB code 5AK1) as the search model. The refinement was done in RefMac5 (27), and COOT (26) was used for model (re)building. The final model were validated using Molprobity (32). The data collection and refinement statistics are listed in Table 2.

**TABLE 2:** **Data collection and refinement statistics** The values in parentheses are for highest resolution shell.

	CtXyl5A_-D_	GH5-CBM6-*Arap*	GH5-CBM6-*Xylp*	GH5-CBM6- (*Araf*-Xyl*p*_4_)
**Data collection**				
Source	ESRF-ID14-1	Diamond I04–1	Diamond I24	Diamond I02
Wavelength (Å)	0.9334	0.9173	0.9772	0.9791
Space group	P2_1_2_1_2	P2_1_2_1_2_1_	P2_1_2_1_2_1_	P2_1_2_1_2_1_
Cell dimensions				
*a*, *b*, *c* (Å)	147.4, 191.7, 50.7	67.1, 72.4, 109.1	67.9, 72.5, 109.5	76.3, 123.2, 125.4
α, β, γ (°)	90, 90, 90	90, 90, 90	90, 90, 90	90, 90, 90
No. of measured reflections	244,475 (29,324)	224,842 (11,281)	152,004 (4,996)	463,237 (23,068)
No. of independent reflections	42246 (5,920)	63,523 (3,175)	42,716 (2,334)	140,288 (6,879)
Resolution (Å)	50.70–2.64 (2.78–2.64)	44.85–1.65 (1.68–1.65)	45.16–1.90 (1.94–1.90)	48.43–1.65 (1.68–1.65)
*R*_merge_ (%)	16.5 (69.5)	6.7 (65.1)	2.8 (8.4)	5.7 (74.9)
CC_1/2_	0.985 (0.478)	0.998 (0.594)	0.999 (0.982)	0.998 (0.484)
*I*/σ*I*	8.0 (2.0)	13 (1.6)	26.6 (8.0)	11.2 (1.6)
Completeness (%)	98.5 (96.4)	98.5 (99.4)	98.6 (85.0)	98.8 (99.4)
Redundancy	5.8 (5.0)	3.5 (3.6)	3.6 (2.1)	3.3 (3.4)

**Refinement**				
*R*_work_/*R*_free_	23.7/27.8	12.2/17.0	12.9/16.1	14.5/19.9
No. atoms				
Protein	5446	3790	3729	7333
Ligand	19	20	20	92
Water	227	579	601	923
B-factors				
Protein	41.6	17.8	15.8	21.0
Ligand	65.0	19.4	24.2	39.5
Water	35.4	38.5	32.2	37.6
R.m.s deviations				
Bond lengths (Å)	0.008	0.015	0.012	0.012
Bond angles (°)	1.233	1.502	1.624	1.554
Protein Data Bank code	5G56	5LA0	5LA1	2LA2

## Author Contributions

A. L. obtained crystals of the GH5-CBM6 complex. L. I. C. analyzed the biochemistry of GH5 subfamilies. J. L. A. B. obtained crystals of CtXyl5A-D. A. J. analyzed the biochemistry of GH5-CBM6 and obtained crystals of GH5-CBM6. A. R. analyzed the biochemistry of GH5-CBM6 products. J. G. performed mass spectrometry. M. P. Y. provided the substrate. B. H. performed analysis of GH5 sequences. C. M. G. A. F. designed the experiments. H. J. G. designed the experiments, analyzed data, and contributed to writing the paper. S. N. solved the structure of CtXyl5A-D and contributed to writing the paper. A. B. used crystallography to solve GH5-CBM6 structures. F. C. analyzed the biochemistry of GH5-CBM6 mutants, obtained crystals of GH5-CBM6, and contributed to writing the paper.

## Supplementary Material

Supplemental Data

## Footnotes

5 The abbreviations used are:

GH: glycoside hydrolase
*Ct*Xyl5A: *C. thermocellum* arabinoxylanase
CBM: non-catalytic carbohydrate binding module
Fn: fibronectin
WAX: wheat arabinoxylan
RAX: rye arabinoxylan
CX: corn bran xylan
HPAEC: high performance anion exchange chromatography
PDB: Protein Data Bank
BX: birchwood xylan
ESI: electrospray ionization.
