# Supplementary material for: The Mechanism by Which Arabinoxylanases Can Recognize Highly Decorated Xylans
Source: J Biol Chem. 2016 Aug 16;291(42):22149–59. doi: 10.1074/jbc.M116.743948 (PMC5063996; doi:10.1074/jbc.M116.743948)
Supplement: Supplemental Data [file supp_291_42_22149__index.html]

The mechanism by which arabinoxylanases can recognise highly decorated xylans — The Mechanism by Which Arabinoxylanases Can Recognize Highly Decorated Xylans — Mechanism of Arabinoxylanase — Supplemental Data 

# The Mechanism by Which Arabinoxylanases Can Recognize Highly Decorated Xylans

## Supplemental Data

- Suppllemental Fig. S1 (.pdf, 50 KB) - Sequence alignment of arabinoxylanases
- Supplemental Table 1 (.pdf, 17 KB) - Primers used to amplify or mutate DNA
