# Supplementary material for: The Mechanism by Which Arabinoxylanases Can Recognize Highly Decorated Xylans
Source: J Biol Chem. 2016 Aug 16;291(42):22149–59. doi: 10.1074/jbc.M116.743948 (PMC5063996; doi:10.1074/jbc.M116.743948)
Supplement: Supplemental Data [file 10.1074_M116.743948_jbc.M116.743948-2.pdf]

TABLE S1 Primers used for cloning and site-directed mutagenesis

Primer Name	Sequence	Restriction Enzyme	Plasmid
*Ct*GH5CBM6 E68AF	CCATACACTTCCACGGCCTGGACGGCGGCAGCT	N/A	pET28b
*Ct*GH5-CBM6 E68AR	AGCTGCCGCCGTCCAGGCCGTGGAAGTGTATGG	N/A	
*Ct*GH5-CBM6 Y92AF	GCAGTACACCTCGCCGCAGAATGC	N/A	pET28b
*Ct*GH5-CBM6 Y92AR	GCATTCTGCGGCGAGGTGTACTGC	N/A	
*Ct*GH5-CBM6 N135AF	GTAATAACCATAGGCGCCGGTGCCAATAAC	N/A	pET28b
*Ct*GH5-CBM6 N135AR	GTTATTGGCACCGGCGCCTATGGTTATTAC	N/A	
*Ct*GH5-CBM6 N139AF	GGCAACGGTGCCAATGCCGGAAATCATAACGCG	N/A	pET28b
*Ct*GH5-CBM6 N139AR	CGCGTTATGATTTCCGGCATTGGCACCGTTGCC	N/A	
*Ac*GH5F	ATTTCTCATATGCATGCAGATCCTCAGCG	NdeI	pET21a
*Ac*GH5R	ATTAATGAATTCCAGCCGAGAACTTGAATCATTTTT TCAGCATACCGC	EcoRI	
*Gp*GH5F	Not required	NheI	pET21a
*Gp*GH5R	Not required	XhoI	
*Vb*GH5F	ATTTATGCTAGCGATGGCACCTTTAGCAATCCGCTT CTGTATGC	NheI	pET28b
*Vb*GH5R	TATTATCTCGAGAGGACGCGGTTCTTCATCACTCAG TGCC	XhoI	
*Vb*GH5D45AF	AGATTATCCGGCCCCTGATATTATTCGTGTTGATGA GGC	N/A	pET28b
*Vb*GH5D45AR	GCCTCATCAACACGAATAATATCAGGGGCCGGATA ATCT	N/A	

N/A: not applicable
