# Supplementary material for: The Mechanism by Which Arabinoxylanases Can Recognize Highly Decorated Xylans
Source: J Biol Chem. 2016 Aug 16;291(42):22149–59. doi: 10.1074/jbc.M116.743948 (PMC5063996; doi:10.1074/jbc.M116.743948)
Supplement: Supplemental Data [file 10.1074_M116.743948_jbc.M116.743948-1.pdf]

```
VbGH5      MCSYSYDGAFWTRFGGIFTLGFDITLATFQGQKFALFCYNTGFESSSGYVDVDSFTFGDT     60
GpGH5      ------------------------------------------------------------     0
CtGH5      -------------MGASIKTSIKIRTVAFVSIIA---------------IALSILSFIPN     32
AcGH5      -----------------MKLSIRIRT-AFAAAIA---------------LVLCSLSLVPN     27

VbGH5      AEQVVTQRGWAVLNEAGTTFVADNGQLLRGPFASTEWGDPPPRENLAKLKELGLNAVHLY     120
GpGH5      ---ALAARGRPRLNG--KTFVADSGVPLRGPFTSTEWTPAVPAANIANMRNYNFNAIHLY     55
CtGH5      RAYASPQRGRPRLNAARTTFVGDNGQPLRGPYTSTEWTAAAPYDQIARVKELGFNAVHLY     92
AcGH5      QVHADPQRGRPYLNSARTTFVGDNGQPLRGPYISTEWTSAAPYDQIARIKNLGFNAVHHY     87
              .   **   **   .***.*.*  ****: ****     *  ::*.:::  :**:* *

VbGH5      AEVFDPDYPAEGSQGPGYSASRIDDMVEMTRELGLYLVMTIGNGANNGDYNRDYIVDFWD     180
GpGH5      AETFDPNYPAAGSQKPGYAATRVDQIVAATKAANMYVVIVLANGANNGKFNLNYAKDFWS     115
CtGH5      AECFDPRYPAPGSKAPGYAVNEIDKIVERTRELGLYLVITIGNGANNGNHNAQWARDFWK     152
AcGH5      AECFDINYPNAGSKSPGYAATEIDKVVERTRELGLYLVMTIGNGANNGNHNTRYAKDFWS     147
           ** **  **  **: ***:...:*.:*  *:   :*:*:.:.******..*  :  ***.

VbGH5      LYAERYADESHVVFEIQNEPVAWTPPYSQA------VLDMEVAAYDTIRSHAPDTPVLLM     234
GpGH5      FYAARYKNETHVIYEIHNEPVQWGPPYISSTQ-SPGAVSMNADCYKIIRAVAPDTPVLLF     174
CtGH5      FYAPRYAKETHVLYEIHNEPVAWGPPYSSSTANPPGAVDMEIDVYRIIRTYAPETPVLLF     212
AcGH5      FYSSRYANETHVLYEIHNEPVAWGPPYSSTTATPTGAVEMNVDVYKTIRANAPKTPVLIF     207
           :*: ** .*:**::**:**** * *** .:      .:.*:   *  **: **.****::

VbGH5      TYAVLSNWSAALSDLSKI---------HDDIDWNNAAVGFHGYAGHEETPPAVEAILEAG     285
GpGH5      TYASIGGGSSAAGAVKDAQSFNTAVFGNANAQWTNEAIAIHGYWGAQGASDAAKALNAAG     234
CtGH5      SYAVFGGKGGAAEALKDIRAFNKAVFGNENAVWTNEAVAFHGYAGWQETTIAVEELLKAG     272
AcGH5      SYSVFGGTGGTTEALKDIQAFNSAVFGKQDAVWTNEAVAFHGYAGWEATSTAVDGLLKAG     267
           :*: :.  ..:   :..          . :  *.* *:.:*** * : :  *.. :  **

VbGH5      YPMFMTEFTTSEW-GYSLDHIDLEMTATLERLGISWVNFLHVPPNFINEAITDPTAYSDI     344
GpGH5      FSVVLTEFAAATSPTSPNGGQDTVLTGFMEQQGVSWLTFLHVPPTGVSGDVTDPNQYTNR     294
CtGH5      YPCFMTEYAGGAW-GSGMGGLDVELTYELERLGVSWLTFQYIPPTGVSDDVTKPEYFSAL     331
AcGH5      YPCFMTEYAGGAW-GSGTGGFDIQLTSELERMGVSWLTFQYIPPSGVSDDVTKPEYFSAL     326
           :  .:**:: .          *  :*  :*: *:**:.* ::**. :.  :*.*  ::

VbGH5      VNRSGLGWVPDFGDWPVQRGVFGNEGLPRETTSNWVNNKLSGTLRVEAEDFDVGRHGVAY     404
GpGH5      MTAAGIGFDRDPGLNAVGGGQAA-------------------------------------     317
CtGH5      VENSGLSWTPDYGNWPAARGVYGNGGLARET-ATWINNFLTGTTRIEAEDFDWGGNGVSY     390
AcGH5      VENAGLSWKPDYGNWPAARGVHGNGGLPRKT-STWVNNFLTGTTRIEAEDFDWGGNDVSF     385
           :  :*:.:  * *   .  *  .

VbGH5      SDRDSVNKGGQYRPEEGVDILSVSDGSDGFAVNSMELDEWLEYTIFVKEPGLYTFRLHYA     464
GpGH5      ------------------------------------------------------------     317
CtGH5      YDTDSVNVGGQYRPDEGVDIEKTSDTGGGYNVGWISEGEWLEYTIRVRNPGYYNLSLRVA     450
AcGH5      YDKDSENKGAQYRLDEAVDIETTKDADGGYNVGWIEDGEWLEYTIWVQHPGYYNLALRVA     445

VbGH5      SAVSSGAIQANFNGVDSTGSITLPSTGGASVWGTVEREVFLDYGQQKVRLTVESGGMMVN     524
GpGH5      ------------------------------------------------------------     317
CtGH5      G-ISGSRVQVSFGNQDKTGVWELPATGGFQTWTTATRQVFLGAGLQKLRINALSGGFNLN     509
AcGH5      N-NSGGSVQVNFGNQDKTGTWVLPVTGGVQTWKTDTRQVFLGSGRQKLRINALSGGFNLN     504

VbGH5      WFELTPVAN    533
GpGH5      ---------    317
CtGH5      WIELSPIST    518
AcGH5      WIELSPVST    513
```

FIGURE S1. Sequence alignment of GH5_34 sub family. The -2* pocket residues are highlighted in yellow and catalytic residues are highlighted in magenta. Alignment generated using Clustal omega.
